# Effects of Aerobic Exercise on Rats with Hyperandrogenic Polycystic Ovarian Syndrome

**DOI:** 10.1155/2021/5561980

**Published:** 2021-08-09

**Authors:** Na Li, Chenghao Yang, Huiyu Xie, Yinghong Liu, Yuanpeng Liao

**Affiliations:** ^1^Department of Sports Medicine and Health, Chengdu Sport University, Chengdu 610041, China; ^2^School of Kinesiology, Shanghai University of Sport, Shanghai 200438, China; ^3^Department of Preventive Medicine, Keck School of Medicine, University of Southern California, Los Angeles 90033, USA

## Abstract

Hyperandrogenism is a key pathologic characteristic of polycystic ovarian syndrome (PCOS), and exercise can alleviate the accompanying inflammation and decrease the high androgen levels, but the mechanism is still unclear, so the purpose of this study is to explore the pathophysiologic characteristics of hyperandrogenic PCOS and the mechanism underlying its amelioration with aerobic exercise. Thirty-two female rats were randomly allocated to a normal control group (NC, *n* = 8), exercise control group (EC, *n* = 8), PCOS group (PC, *n* = 8), and PCOS plus exercise group (PE, *n* = 8). The PC and PE groups were injected with a dehydroepiandrosterone (DHEA) solution to induce the hyperandrogenic PCOS rat model. The EC and PE groups underwent a Masashi swimming protocol (120 min per session, 6 days/week, for 15 days). Results indicated that the concentrations of leptin (LP) in the EC group were significantly lower than those in the NC group (*p* < 0.05). Compared with the NC group, the levels of testosterone (T), estradiol (E_2_), follicle-stimulating hormone (FSH), LP, anti-Müllerian hormone (AMH), tumor necrosis factor-alpha (TNF-*α*), interleukin-6 (IL-6), and free fatty acids (FFA) were all significantly augmented in the PC group (all *p* < 0.05). In addition, compared with the NC group, the levels of adiponectin (ADP) were significantly decreased (*p* < 0.05), and the expression of aromatase cytochrome P450 (P450arom) in ovarian tissue was significantly elevated in the PC group (*p* < 0.05). The levels of T, FSH, LP, and FFA were also significantly increased in the PE group (*p* < 0.05). Compared with the PC group, the levels of T and LP in the PE group were significantly diminished (*p* < 0.05), and the levels of ADP were significantly increased in the PE group (*p* < 0.05). T was positively correlated with E_2_, FSH, AMH, LP, TNF-*α*, IL-6, and FFA levels, while ADP was negatively correlated with LP and E_2_. These results showed that hyperandrogenism, chronic low-grade inflammation, and leptin resistance may interact to influence the occurrence and development of PCOS. Aerobic exercise can alleviate the internal inflammation by relieving leptin resistance and may mitigate the sex hormone disorder and hyperandrogenism in rats with PCOS by affecting the hypothalamic-pituitary-ovarian axis.

## 1. Introduction

Polycystic ovary syndrome (PCOS) is a common endocrine and metabolic disorder with a global prevalence of 4–21% [1, 2]. The clinical manifestations of PCOS include ovulation failure, menstrual disorders, polycystic ovaries, obesity, and hyperandrogenism associated with hirsutism, acne, and alopecia [3, 4]. The pathogenesis of PCOS still remains unclear, although recent studies suggest that it is related to genetic, epigenetic, and environmental factors [5]. The hypothalamic-pituitary-gonadal (HPG) axis regulates the normal gonadal function through the feedback mechanism, and patients with PCOS usually have obvious dysfunction of the hypothalamic-pituitary-ovarian (HPO) axis [6]. Abnormal neurotransmitter-related synthetase and receptors in the central nervous system of PCOS patients lead to hypersecretion of gonadotropin-releasing hormone (GnRH) pulse in the hypothalamus, which further causes increased release frequency and amount of luteinizing hormone (LH). However, the secretion of follicle-stimulating hormone (FSH) is normal or slightly lower, thus increasing the ratio of LH/FSH in serum [7]. A relatively low level of FSH and development and maturation disorders of the follicle eventually leads to anovulation, polycystic change of the ovarian. Excessive LH can promote the proliferation of ovarian interstitial and follicular membrane cells and also excessive androgen secretion, eventually leading to hyperandrogenism.

Hyperandrogenism is a key pathophysiologic characteristic of PCOS [8], and the injection of large doses of androgens is generally used to induce an experimental model of hyperandrogenic PCOS in rats [9]. PCOS is considered to be associated with chronic low-grade inflammation and increased androgen levels, and these changes can easily lead to adipocyte hypertrophy [10]. This in turn releases various adipocytokines involved in the regulation of lipid metabolism, inflammation, and reproductive processes [11], resulting in increased serum inflammatory markers such as tumor necrosis factor-alpha (TNF-*α*), interleukin-6 (IL-6), and C-reactive protein (CRP) [12]. These upregulated markers then precipitate chronic low-grade inflammation and ultimately lead to a series of sex hormone disorders. Other researchers also found that due to obesity in PCOS patients, the increased circulating leptin (LP) levels within the body led to leptin resistance, which can also significantly promote chronic low-grade inflammation [13]. Hence, we herein speculated that in the pathophysiologic process of PCOS, hyperandrogenism, leptin resistance, and chronic low-grade inflammation may form a vicious cycle, but the relationships among the three are not completely clear.

Although PCOS is difficult to cure completely, it can be effectively controlled. The American Society for Reproductive Medicine (ASRM) and European Society of Human Reproduction and Embryology (ESHRE) published the International Evidence-Based Medicine Guidelines for the Evaluation and Management of Ovarian Syndrome in 2018, which recommended a combination of medication and lifestyle interventions for the long-term management of PCOS patients [14]. Recent studies have shown that antioxidant drugs such as *Galega officinalis* extract, celery extract, and cinnamon extract have a protective effect on ovaries and can effectively improve ovarian dysfunction caused by oxidative stress [15, 16]. Optimal exercise has also been hypothesized to prevent and treat numerous metabolic diseases such as PCOS [17, 18]. Studies have shown that exercise may promote adipose tissue resistance to the chronic inflammation caused by obesity through signal pathways such as insulin and transforming growth factor-beta (TGF-*β*) [19] and to downregulate inflammatory responses and inflammation-related genes that are activated by obesity (such as TNF-*α*, IL-6, and their receptors [20]).

Thus, the purpose of this study was to explore the effect of exercise intervention on rats with hyperandrogenic PCOS by investigating their inflammatory status and hormonal levels. We also wished to further investigate how chronic low-grade inflammation, hyperandrogenism, and leptin resistance interacted to lead to the occurrence and development of PCOS and the possible underlying mechanism by which aerobic exercise alleviated hyperandrogenism in PCOS. With this study, we expect to provide a foundation for the clinical treatment of PCOS and to prescribe exercise formulations for patients with PCOS.

## 2. Materials and Methods

### 2.1. Animals

Thirty-two specific pathogen-free (SPF) female Sprague-Dawley (SD) rats (20 days old, 64 ± 14 g) were purchased from Chengdu Dashuo Biological Technology Co., Ltd. (Chengdu, China) (SYXK 2018-24), fed in the animal laboratory of Chengdu Sports University (room temperature, 22–24°C; relative humidity, 60–75%; 12 h/12 h, light/dark cycle), and were provided chow ad libitum. The weights of the rats were numbered from low to high, and then, the rats were randomly allocated into a normal control group (NC, *n* = 8), exercise control group (EC, *n* = 8), PCOS control group (PC, *n* = 8), and PCOS exercise group (PE, *n* = 8). After 3 days of adaptive feeding, the PC and PE groups were injected with dehydroepiandrosterone (DHEA) solution (Beijing Solarbio Technology Co., Ltd., Beijing, China; concentration, 30 mg/mL; injection volume, 0.2 ml/100 g) subcutaneously into the back of the neck every morning for 20 days to induce a hyperandrogenic PCOS rat model [21]. Our study procedures were approved by the Animal Research Committee of Chengdu Sports University (permit number SYXK 2018-211), and we followed them assiduously.

### 2.2. Analysis of Estrous Cycles

Microscopic evaluation of the cell types present in vaginal smears has been used for decades to distinguish the stages of the estrous cycle in rats and as an indicator of the functional status of the HPO axis [22]. We took vaginal smears from the 15th day of model preparation and observed them microscopically between 8 : 00 and 9 : 00 am every day to allow evaluation of the estrous cycle as judged by cell type and number [23]. (1) Proestrus was characterized by the presence of small, round, nucleated epithelial cells of relatively uniform appearance and size; (2) estrus by the presence of predominately anucleated, keratinized epithelial cells; (3) metestrus by a combination of anucleated, keratinized epithelial cells and neutrophils; and (4) diestrus as a combination of neutrophils, with small and large nucleated epithelial cells, and neutrophils were usually higher in number relative to epithelial cells, with smears sometimes being exclusively neutrophilic. If the rats in the PC group are always in diestrus, with a significant increase in serum testosterone (T), along with numerous atretic follicles, enlarged stroma, and the existence of cystic follicles, we could identify that the rats in the PC group had hyperandrogenism, ovulation failure, and cystic ovarian. These syndromes were consistent with the main characteristics of the diagnosis of PCOS. These also indicated the successfully established animal model. At the same time, if the rat vaginal smear does not show estrous cycle, it is discarded [24].

### 2.3. Exercise Protocol

From the second day after the successful establishment of the hyperandrogenic PCOS rat model, the EC and PE groups underwent the Masashi swimming protocol [25, 26]: the rats were placed in a pail with a water depth of 50 cm at a water temperature of 35°C for free swimming without any load. The exercise protocol was 120 min/per session, 6 days/week, for 15 days. We regularly placed the cage next to the pail during exercise to eliminate the influence of environmental noise. The four groups were fed identical diets. The rats in each group were sacrificed by injection with 3% pentobarbital after 24 h of fasting at 8 : 00 pm on the day when the last exercise was completed, and all experimental indicators were then analyzed.

### 2.4. Serum Analysis

Blood samples (5 mL) were taken from the abdominal aorta and centrifugated (3000 r/min) for 8 min, and the supernatant was aspirated. The concentrations of anti-Müllerian hormone (AMH), T, estradiol (E_2_), FSH, LP, TNF-*α*, IL-6, and free fatty acids (FFA) in rat serum were measured with an enzyme-linked immunosorbent assay (ELISA) kit (Shanghai Zhuocai Biotechnology Co., Ltd., Shanghai, China).

### 2.5. Morphologic Analysis of the Ovary

After rats were sacrificed, we dissected out both ovaries and separated them. One ovary was fixed in 4% paraformaldehyde, embedded in paraffin, and sectioned at 4 *μ*m. After hematoxylin and eosin staining (H&E, Beijing J&K Scientific Technology Co., Ltd., Beijing, China), the morphology of the ovary was observed with a BA200Digital digital 3-eye video microscopy system (MOTIC China Group Co., Ltd., Xiamen, China). Each slice was observed to determine the gross lesions (at ×100 magnification). Then, we observed the selected area to determine the specific lesions (at ×100 magnification and ×400 magnification). The contralateral ovary was quick-frozen in liquid nitrogen and stored at −80°C for later use.

### 2.6. Immunohistochemistry of Aromatase Cytochrome P450

Localization and intensity level of aromatase cytochrome P450 (P450arom) staining in rat ovarian tissue were determined immunohistochemically. Sections were deparaffinized, hydrated, and then pretreated in a microwave (antigen retrieval). Endogenous peroxidase activity was inhibited with 3% methanol hydrogen peroxide. Normal goat serum (10%, Beijing ZsBio Technology Co., Ltd., Beijing, China) was utilized at room temperature for 20 minutes, to block the nonspecific binding sites. P450arom immunodetection was carried out using a polyclonal rabbit anti-rat (1 : 100, Beijing ZsBio Technology Co., Ltd., Beijing, China) at 4°C overnight. Then, a biotinylated goat-anti-rabbit (1 : 100, Beijing ZsBio Technology Co., LTD., Beijing, China) was applied for 30 min at 37°C. Immunoreactivity was visualized by using the diaminobenzidine chromogen (DAB) (Beijing ZsBio Technology Co., Ltd., Beijing, China), and finally, slices were counterstained with hematoxylin (Beijing J&K Scientific Technology Co., Ltd., Beijing, China). Antibodies were replaced with PBS for a negative control. For evaluation, a semiquantitative analysis of the staining results was achieved using the Image-Pro Plus 6.0 image quantitative analysis system. Positive results for P450arom were brown or yellow particles stained among granular cells. The positive area and the optical density (OD) values in 3 high-power optical fields (×400 magnification) of every slice were measured. The immune positive area index (positive area/total area × OD) values of P450arom were calculated in every high-power optical field.

### 2.7. Statistical Analysis

Statistical analysis was performed using SAS 9.4 software (SAS Institute Inc., Cary, NC, USA). All data were graphed using GraphPad Prism 8.0 software (GraphPad Software Inc., San Diego, CA, USA). Descriptive data were depicted as means ± standard deviation (SD). Residual analysis was performed to assess normality and homoscedasticity. Data were analyzed using one-way ANOVA followed by Tukey's test. Interrelationships among all indicators were evaluated with the Pearson correlation coefficient (*r*). All results were considered significantly at a *p* value < 0.05.

## 3. Results

### 3.1. Rat Estrous Cycle Analysis of Each Group

**Figure 1:**
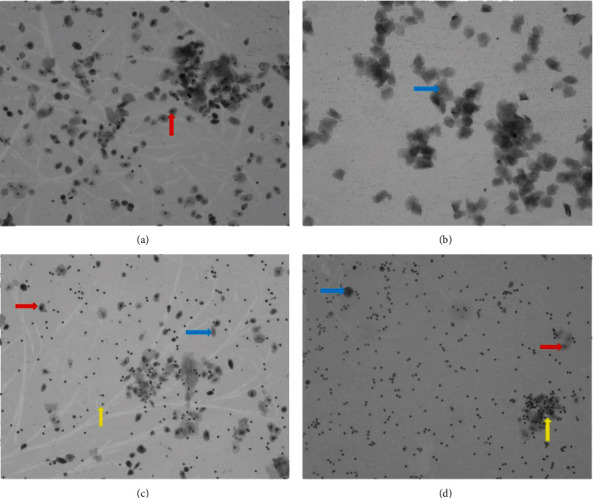
Changes in the estrous cycles of rats (×200). (a) Proestrus, a large number of small nucleated epithelial cells found individually and in cohesive clusters (red arrow). (b) Estrus, a large number of anucleated epithelial cells (blue arrow). (c) Metestrus, neutrophils interspersed or clumped (yellow arrow) among the nucleated (black arrow) and anucleated epithelial cells (blue arrow). (d) Diestrus, predominately neutrophils (yellow arrow) that are mixed with a few epithelial cells (black arrow and blue arrow).

Rat vaginal smears showed that the estrous cycles of the NC and EC groups were similar and relatively stable and regular (Figures 1(a)–1(d)). However, rats in the PC and PE groups were in constant diestrus (Figure 1(d)), and the vaginal smears were predominately composed of neutrophils that were mixed with a few epithelial cells. This successfully validated the PCOS rat model. However, from the 8th day of exercise intervention, the estrous cycles of rats in the PE group gradually returned to normal and became more regular and stable, which was similar to the estrous cycles of the NC group.

### 3.2. Pathologic Analysis of Rat Ovaries in Each Group

**Figure 2:**
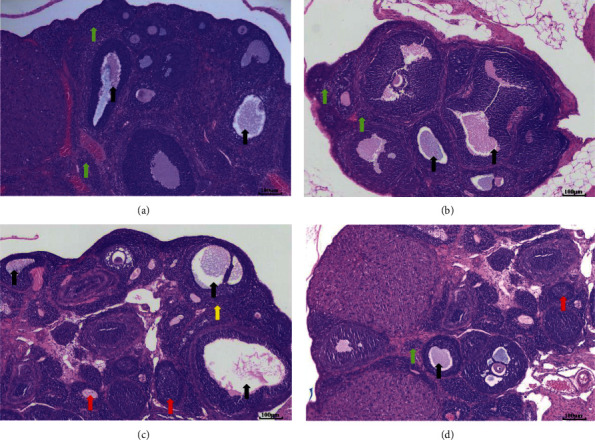
Histologic sections of rat ovaries (×100). (a) Normal control group (NC) with various stages of follicles. (b) Exercise control group (EC) with several antral and atretic follicles. (c) PCOS group (PC) with numerous atretic follicles with enlarged stroma and the presence of cystic follicles. (d) PCOS plus exercise group (PE) with numerous antral follicles, several atretic follicles, and several corpora lutea. Black arrow, antral follicles; red arrow, atretic follicle; green arrow, corpus luteum; yellow arrow, enlarged stroma.

Ovarian morphology of rats in the NC group showed developing follicles at various stages, with a reduced number of atretic follicles, and the layers of granulosa cells were thickened and neatly arranged (Figure 2(a)). Several antral and atretic follicles can be seen in the EC group (Figure 2(b)). Many atretic follicles with enlarged stroma and cystic follicles can also be readily observed in the PC group (Figure 2(c)). Many antral follicles, several atretic follicles, and several corpora lutea were noted in the PE group (Figure 2(d)).

### 3.3. Analysis of Serum from Rats of Each Group

**Figure 3:**
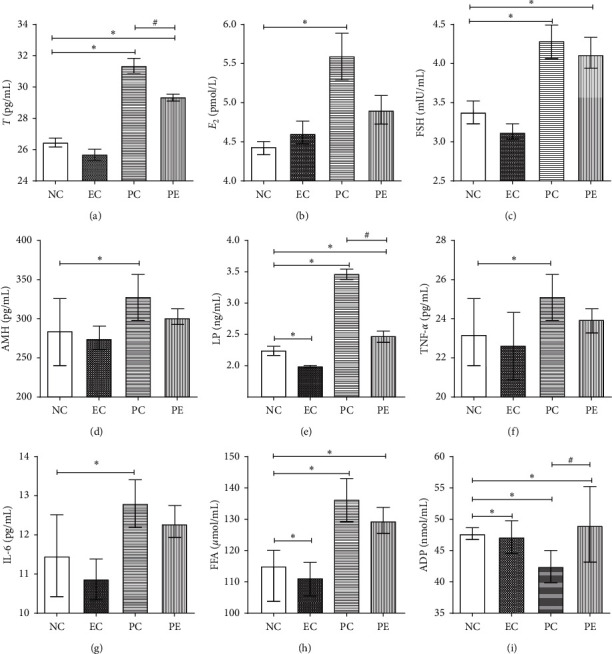
Serum analysis of rats in each group. Abbreviations are the same as in Table 1. ^*∗*^Compared with the NC group, *p* < 0.05. ^#^Comparison between PE and PC groups, *p* < 0.05.

Compared with the NC group, the levels of LP significantly decreased in the EC group (*p* < 0.05), while other indicators showed no statistically significantly differences between the NC and EC groups (*p* > 0.05). Compared with the NC group, there was a significant increase in the levels of T, E_2_, FSH, LP, AMH, TNF-*α*, IL-6, and FFA in the PC group (*p* < 0.05), while the levels of ADP were significantly decreased (*p* < 0.05). The levels of T, FSH, LP, and FFA were significantly elevated in the PE group (*p* < 0.05), while the other indicators showed no statistically significant differences between the NC and PE groups (*p* > 0.05). Compared with the PC group, the levels of T and LP in the PE group were significantly diminished (*p* < 0.05), and the levels of ADP increased significantly (*p* < 0.05), while the other indicators showed no statistically significant differences between the PC and PE groups (*p* > 0.05) (Table 1, Figure 3).

### 3.4. Expression of P450arom in Rat Ovaries

**Figure 4:**
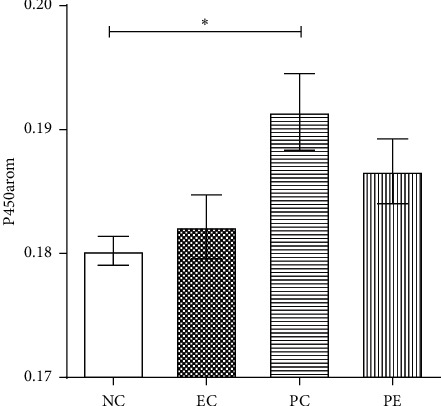
Mean immunohistochemical density of P450arom in ovarian of rats in each group. NC, normal control group; EC, exercise control group; PC, PCOS group; PE, PCOS plus exercise group; P450arom, P450 aromatase. ^*∗*^Compared with the NC group, *p* < 0.05. ^#^Comparison between PE and PC groups, *p* < 0.05.

**Figure 5:**
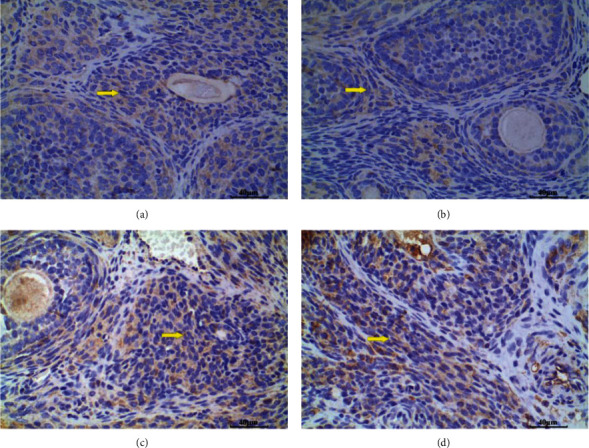
Ovarian micrograph of P450arom expression by immunohistochemistry in rats of each group (×400). (a) Normal control group (NC); (b) exercise control group (EC); (c) PCOS group (PC); (d) PCOS plus exercise group (PE). The substrate was white; the negative cells are presented in blue, and the positive cells are presented in yellow or brownish yellow. The yellow arrow indicates the area of positive expression. Compared with the NC group, the P450arom mean density in the ovarian tissue of the PC group was significantly enhanced.

Compared with the NC group, the expression of P450arom in the PC group increased significantly (*p* < 0.05), but we observed no difference between the EC and PE groups (*p* > 0.05). There was also no significant difference in expression of P450arom between PC and PE groups (*p* > 0.05) (Figures 4 and 5).

### 3.5. Interrelationship Analysis among All Indicators in Rats

**Figure 6:**
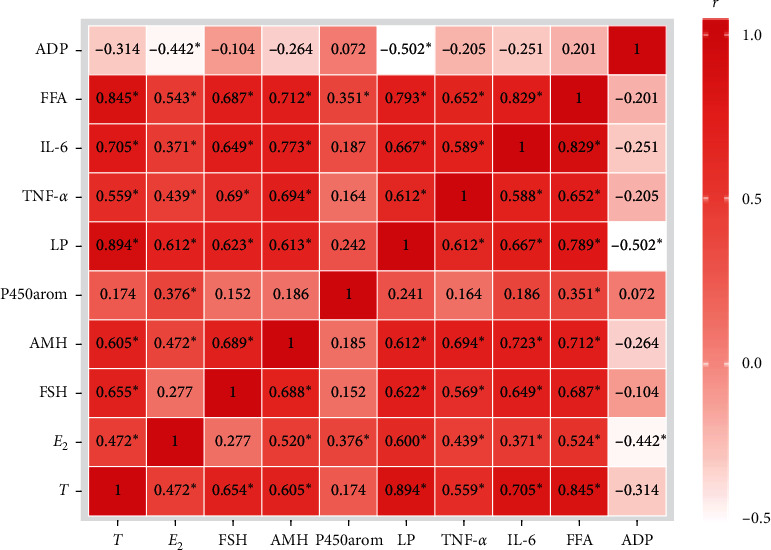
Interrelationship analysis among all indicators. The Pearson correlation coefficient *R* value is shown in the heatmap. NC, normal control group; EC, exercise control group; PC, PCOS group; PE, PCOS plus exercise group; T, testosterone; E_2_, estradiol; FSH, follicle-stimulating hormone; AMH, anti-Müllerian hormone; LP, leptin; TNF-*α*, tumor necrosis factor-alpha; IL-6, interleukin-6; FFA, free fatty acid; ADP, adiponectin; P450arom, P450 aromatase. ^*∗*^*P* < 0.05.

Pearson correlation results (Figure 6) showed that T was positively correlated with E_2_, FSH, AMH, LP, TNF-*α*, IL-6, and FFA (*p* < 0.05) and that E_2_ was positively correlated with AMH, LP, P450arom, TNF-*α*, IL-6, and FFA (*p* < 0.05), but it was negatively correlated with ADP (*p* < 0.05). FSH was positively correlated with AMH, LP, TNF-*α*, and IL-6 (*p* < 0.05); AMH was positively correlated with LP, TNF-*α*, IL-6, and FFA (*p* < 0.05); LP was positively correlated with TNF-*α*, IL-6, and FFA (*p* < 0.05), but negatively correlated with ADP (*p* < 0.05); P450arom was positively correlated with FFA (*p* < 0.05); TNF-*α* was positively correlated with IL-6 and FFA (*p* < 0.05); and IL-6 was positively correlated with FFA (*p* < 0.05).

## 4. Discussion

In the present study, we demonstrated that rats with hyperandrogenic PCOS induced by continuous injection of large doses of DHEA manifested obvious inflammation and leptin resistance and that regular aerobic exercise under the Masashi swimming protocol [25] (which is a commonly used exercise protocol in animal models) ameliorated the inflammation, alleviated leptin resistance, improved the sex hormone metabolic disorder caused by hyperandrogenism, and promoted ovulation.

The pathogenesis of PCOS is still not clear. Previous studies showed that the key pathogenesis of PCOS is the imbalance of related gonadotropin secreted by the HPO axis. The abnormalities in the pulsatile release of GnRH might underlie the development of PCOS [27]. Due to the increased sensitivity of the pituitary to GnRH, LH is excessively produced, stimulating the ovarian stroma and follicular membrane cells to produce excessive androgen. A high level of androgen in the ovary will inhibit the maturation of follicles, so that dominant follicles cannot form. However, small follicles in the ovary can still secrete E_2_ equivalent to the level of the early follicular stage. In addition, androstenedione can be converted to estrone under the action of peripheral tissue aromatase, leading to the high content of estrone in the serum. The sustained production of estrone and a certain level of E_2_ act on the hypothalamus and pituitary glands and play a positive feedback role on producing LH, increasing the pulse amplitude and frequency of LH production. Estrogen has a negative feedback effect on FSH secretion, making the FSH level relatively lower, which cause the ratio of LH/FSH to increase [28, 29]. A high level of LH enhances hypersecretion of androgens in theca cells in ovarian follicles. However, a low level of FSH can impair follicular development and lead to the formation of nondominant follicles, thus promoting a vicious cycle of excessive androgens and continuous anovulation [30].

Hypothalamic dysfunction is a critical factor underlying metabolic syndrome and related diseases [31–34]. In our study, we found that the levels of proinflammatory cytokines in the PC group were significantly higher relative to those in the NC group, while the levels of anti-inflammatory cytokines were lower compared to the NC group, and we observed significant leptin resistance in the PC group, consistent with the results of previous studies. Studies have shown that typical activation of proinflammatory signals occurs in the hypothalamus under conditions of endocrine disorders and metabolic syndrome, which promotes the secretion of inflammatory factors by adipose tissue and causes “hypothalamic microinflammation” [35], affecting the LP pathway and leading to leptin resistance [36–38]. Leptin resistance was thought to be involved in the regulation of reproductive function through the HPO axis [39], causing ovarian dysfunction [40], and some studies have suggested that LP is related to the occurrence of PCOS [41, 42]. In addition, excessive androgen secretion promotes the conversion of T to the more active dihydrotestosterone (DHT), further promotes the release of more proinflammatory cytokines (TNF-*α*, IL-6, and FFA) in adipose tissue, and inhibits the secretion of the anti-inflammatory cytokine ADP [43], leading to “hypothalamic microinflammation.” ADP inhibits the synthesis of testosterone substrates (e.g., androstenedione) [44], and the decrease in ADP increases androgen levels.

Our correlation analysis results showed that LP levels were positively correlated with FSH, E_2_, and T levels, suggesting that hyperandrogenism may lead to the increase in LP through some signal pathways and that leptin resistance was one of the principal causes of the disorder in sex hormones in PCOS patients. After LP binds to specific receptors in the hypothalamus, it inhibits the secretion of neuropeptide Y (NPY), promotes the release of GnRH (further promoting the release of LH and FSH), and stimulates ovarian follicular cells to synthesize androgens [45], thereby inhibiting the maturation of follicles and affecting the endocrine and reproductive systems. FSH receptors are only found on the surface membranes of ovarian granulosa cells, and these cells when stimulated by FSH then activate P450arom. P450arom is the rate-limiting enzyme in the last step of estrogen synthesis, and defects in its enzymatic activity constitute the primary cause of infertility in women [46], as androgens are aromatized to E_2_ under the catalysis of P450arom [47]. A previous study showed that leptin resistance inhibited the expression of P450arom, prevented the transformation of androgen to estrogen, and then inhibited the secretion of estrogen [48]. Our correlation analysis results also showed that AMH was positively correlated with T, FSH, E_2_, and the expression of P450arom. T promotes the initial growth of primary follicles and the proliferation of theca and granulosa cells, and in the case of high T levels, follicles may produce more AMH, thus inhibiting follicular recruitment and follicular growth, eventually leading to dysfunction in the selection of dominant follicles and in ovulatory disorders. AMH inhibits the expression of P450arom by reducing the sensitivity of granulosa cells to FSH, and this leads to the overall accumulation of androgens and the inhibition of estrogen synthesis [49]. High androgen levels also promote the production of AMH, thus forming a vicious cycle. However, we found that the levels of T, FSH, and E_2_ in the PC group were significantly higher than those in the NC group, which may have been caused by enhanced binding of FSH to its receptor on granulosa cells. This led to the overexpression of P450arom in ovarian tissues and augmented the levels of androgen, which in turn led to the increase in the precursor substrates needed to synthesize estrogen, resulting in a relative increase in the levels of E_2_.

**Figure 7:**
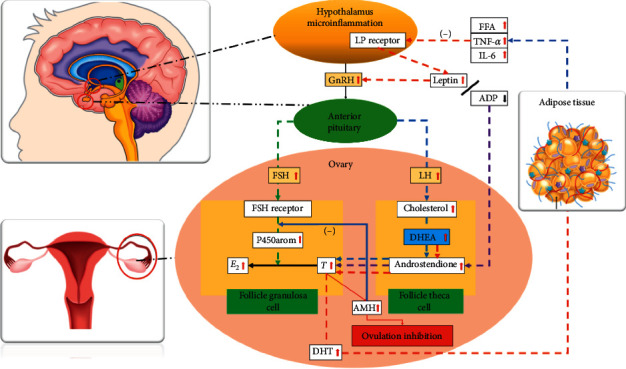
Pathophysiologic mechanism underlying hyperandrogenic PCOS. E_2_, estradiol; FSH, follicle-stimulating hormone; AMH, anti-Müllerian hormone; LP, leptin; TNF-*α*, tumor necrosis factor-alpha; IL-6, interleukin-6; FFA, free fatty acid; ADP, adiponectin; P450arom, P450 aromatase; DHEA, dehydroepiandrosterone; DHT, dihydrotestosterone.

Based on the aforementioned results, we hypothesize that hyperandrogenism, chronic low-grade inflammation, and leptin resistance interact to form a vicious cycle. The underlying mechanism leading to ovulatory disorders with PCOS is shown in Figure 7.

Oxidative stress is a state of imbalance between oxidation and antioxidation in the body. Studies have found that hyperandrogenism can increase the body's sensitivity to oxidative stress, resulting in the accumulation of reactive oxygen species (ROS), reactive nitrogen species (RNS), and oxidized lipids in the body. Furthermore, it causes ovarian atrophy, impaired follicular development, maturation disorders, follicular atresia, and other structural and functional abnormalities, leading to ovulation disorders. At the same time, some studies have proposed that oxidative stress can enhance the expression of androgen-related synthase in the ovary, stimulate the production and release of androgen, which finally leads to the occurrence of PCOS. Drugs with antioxidant properties, such as *Panax ginseng* extract, coenzyme Q10, and hydroalcoholic extract of *Olea europaea*, not only protect ovarian from damage but also significantly improve follicular function and follicular survival rate, which eventually improve ovarian function [50–52]. In addition, exercise has been proved to inhibit oxidative stress and inflammation, strengthen the body's antioxidant defensive system, and improve the function of organs and tissues. The interaction between oxidative stress and hyperandrogenism and also its mechanism has not been fully elucidated, which needs to do some further study.

The effects of exercise on immune function are related to the intensity of exercise [53]. Regular, moderate-intensity physical exercise enhances the body's immune function and strengthens its resistance to infectious diseases [54]; however, after repeated high-intensity exercise or long-term, high-load training, investigators have uncovered subclinical and clinical infections in the body due to “temporary” or “cumulative” immunosuppression [20]. Studies have confirmed that low-to-moderate intensity aerobic exercise (e.g., brisk walking and swimming) can increase the ovulatory and pregnancy rates of women with PCOS and improve menstrual cyclicity, body composition, and metabolism, but that it exerts no effect on hirsutism or acne caused by hyperandrogenism [55].

Adipose tissue can synthesize and secrete important adipose cytokines such as LP, TNF-*α*, IL-6, and ADP; participate in the regulation of glucose metabolism, lipid metabolism, and inflammation; and play a key role in the occurrence and development of metabolic syndrome [56]. Studies have shown that exercise may stimulate adipose tissue to exert anti-inflammatory effects through signal pathways such as insulin and TGF-*β* and downregulate the levels of TNF-*α*, IL-6, and LP [20]. The effect of exercise on the improvement of the endocrine metabolism is related to central and peripheral leptin resistance. Studies have shown that exercise can reduce peripheral leptin resistance by increasing muscle mass and brown adipose tissue and also inhibit the expression of skeletal muscle and hepatic leptin signal transduction proteins [57]. In addition, exercise can also reduce central leptin resistance by regulating the neuronal activity of the hypothalamus, regulating the expression of leptin signal transduction protein in the hypothalamus, and increasing the expression of IL-6 in the hypothalamus [58].

The ADP/LP ratio is considered to be a functional biomarker of adipose tissue inflammation from recent studies, and a reduced ADP/LP ratio was suggestive of dysfunctional adipose tissue and elevated systemic inflammation [59]. Our correlation analysis results showed that LP was negatively correlated with ADP and positively correlated with proinflammatory factors such as TNF-*α*, IL-6, and FFA, and this is consistent with previous results and supports the contention that high LP levels are closely related to inflammation [60, 61]. An attenuation in inflammation can upregulate the levels of ADP, thus inhibiting the synthesis of androstenedione, reducing the levels of serum androgen, and alleviating hyperandrogenism. Our results also showed that after aerobic exercise intervention, the levels of LP in the EC group were significantly lower than those in the NC group, but that the levels of T were still high. This may be related to the short intervention period of our experiment, and we thus failed to achieve the optimal interventional effect. However, compared with the PC group, the levels of T and LP in the PE group decreased significantly, while the levels of ADP increased significantly, which was consistent with the results of previous studies.

The effects of exercise on T were also related to the duration and intensity of exercise. Studies showed that short-term exercise or a short-term, moderate-intensity exercise significantly increased T levels [62] and that long-term, heavy-load exercise diminished T levels [63]. We posit that exercise leads to a decrease in T levels because (1) high-intensity exercise affects the HPO axis and eventually attenuates T; (2) testicular stromal cells manifest reduced androgen-receptor binding and decreased affinity for LH, thus hindering the synthesis of T; and (3) abnormal cholesterol metabolism affects the secretion of T by testicular stromal cells [64]. Numerous studies have shown that there is a correlation between LP and T [65, 66], and we herein suggest that after aerobic exercise lessens leptin resistance in rats with hyperandrogenic PCOS, that LP may modulate the levels of T through the HPO axis. Owing to the diminution in serum LP levels, inhibited release of GnRH by the hypothalamus, and reduced FSH in serum, we postulate that the expression of P450arom in ovarian tissue was lessened, reducing the expression of P450arom in ovarian tissue and ultimately reducing the levels of androgen in PCOS rats. However, owing to the reduction in androgen substrate, serum estrogen levels in rats were also reduced commensurately. We additionally found that compared with the NC group, although the levels of T, FSH, LP, and FFA in the PE group were still higher, there were no statistically significant differences in serum E_2_ or related inflammatory factors in the ovarian tissue, suggesting that aerobic exercise improves chronic low-grade inflammation in rats with PCOS.

We acknowledge several limitations to the present study. First, the LEE index (body weight (g)^1/3^ × 10/body length (mm)) is an effective indicator used to evaluate the degree of obesity in adult rats [67]. However, in our study, we only determined the weights of rats before the experiment, and thus, it was difficult to ascertain whether the hyperandrogenic PCOS rats were obese and difficult to analyze whether obesity was related to inflammation and leptin resistance. Second, we used a common aerobic exercise protocol for rats and demonstrated that only some indicators showed significant improvement, which may be related to the shorter intervention period (15 days). Therefore, future studies need to explore more about the effects on PCOS after different exercise forms, exercise intensity, and exercise cycles. Second, further studies can discuss more about the influence of exercise intervention for hyperandrogenic PCOS on oxidative stress state and ovarian tissues' structure or function.

## 5. Conclusions

Chronic low-grade inflammation and leptin resistance may be the key pathophysiologic characteristics of hyperandrogenic PCOS. Hyperandrogenism, chronic low-grade inflammation, and leptin resistance may thus interact to affect the occurrence and development of PCOS. We showed that aerobic exercise can then ameliorate the internal inflammatory state, further mitigate leptin resistance, and improve the sex hormone disorder and hyperandrogenism characteristic of PCOS rats by modulating the HPO axis.

## Figures and Tables

**Table 1:** Serum analysis of rats in each group (mean ± SD).

	NC	EC	PC	PE
T (pg/mL)	26.51 ± 0.77	25.83 ± 0.83	31.55 ± 1.48^*∗*^	29.46 ± 0.89^*∗*#^
E_2_ (pmol/L)	4.41 ± 0.23	4.61 ± 0.42	5.58 ± 0.79^*∗*^	4.90 ± 0.49
FSH (mlU/mL)	3.35 ± 0.37	3.11 ± 0.27	4.25 ± 0.59^*∗*^	4.10 ± 0.56^*∗*^
AMH (pg/mL)	284.37 ± 40.93	275.42 ± 13.77	327.56 ± 29.40^*∗*^	302.58 ± 9.60
LP (pg/mL)	2.24 ± 0.07	1.98 ± 0.01^*∗*^	3.45 ± 0.64^*∗*^	2.47 ± 0.08^*∗*#^
TNF-*α* (pg/mL)	23.18 ± 1.37	22.59 ± 1.64	25.14 ± 1.04^*∗*^	23.87 ± 0.68
IL-6 (pg/mL)	11.44 ± 1.25	10.87 ± 0.51	12.80 ± 0.59^*∗*^	12.29 ± 0.39
FFA (*μ*mol/L)	111.86 ± 7.26	111.12 ± 5.32	135.74 ± 6.83^*∗*^	129.17 ± 4.05^*∗*^
ADP (nmol/L)	47.75 ± 0.91	47.26 ± 2.59	42.44 ± 2.59^*∗*^	49.13 ± 6.03^#^

NC, normal control group; EC, exercise control group; PC, PCOS group; PE, PCOS plus exercise group; T, testosterone; E_2_, estradiol; FSH, follicle-stimulating hormone; AMH, anti-Müllerian hormone; LP, leptin; TNF-*α*, tumor necrosis factor-alpha; IL-6, interleukin-6; FFA, free fatty acid; ADP, adiponectin. ^*∗*^Compared with the NC group, *p* < 0.05. ^#^Comparison between PE and PC groups, *p* < 0.05.

## Data Availability

The data used to support the findings of this study are available from the corresponding author upon request.
